# Proportion of hyperemesis gravidarum and associated factors among pregnant women admitted into the obstetrics ward at Akesta general hospital, North East Ethiopia

**DOI:** 10.1371/journal.pone.0281433

**Published:** 2023-02-06

**Authors:** Kassaye Demewez Adane, Aregash Abebayehu Zerga, Fikre Bayu Gebeyehu, Fanos Yeshanew Ayele

**Affiliations:** 1 School of Public Health, College of Medicine and Health Science, Wollo University, Dessie, Ethiopia; 2 Department of Anatomy, School of Medicine, College of Medicine and Health Science, Addis Ababa University, Addis Ababa, Ethiopia; University of the Witwatersrand Faculty of Health Sciences / Pan African University, SOUTH AFRICA

## Abstract

**Introduction:**

Hyperemesis gravidarum is a condition of intractable vomiting during pregnancy that leads to fluid and electrolyte imbalance, nutrition deficiency and weight loss often requiring hospital admission. Approximately 0.3%-10.8% of pregnant women experience nausea and vomiting during the first trimester of pregnancy. It has been associated with both maternal and fetal morbidity. There is limited evidence about the proportion of hyperemesis gravidarum and associated factors in the study area.

**Objective:**

The aim of this study was to determine the proportion of hyperemesis gravidarum and associated factors among pregnant women admitted into the obstetric ward at Akesta General Hospital, South Wollo Zone, Ethiopia.

**Methods:**

This is hospital-based cross-sectional study of 355 pregnant mothers in Akesta general hospital in northeast Ethiopia from September 1/2018- to August 30 /2020. A simple random sampling technique was used to select the patient card from the whole admission of pregnant women cards during the study period. The diagnosis of hyperemesis gravidarum include persistent vomiting not related to other causes, an objective measure of acute starvation, electrolyte abnormalities and acid-base disturbances, as well as weight loss. The data analysis was done using SPSS version 25. Bivariable and multivariable binary logistic regression analysis was conducted to identify factors associated with hyperemesis gravidarum. Adjusted odds ratio (AOR) with 95% confidence intervals (CI) was reported to show the strength of the association. Statistical significance was stated at P-value < 0.05.

**Results:**

The proportion of hyperemesis gravidarum was 11.3%. Women with previous history of hyperemesis gravidarum AOR (95%CI) = 10.9[2.46, 48.44], previous history of urinary tract infection AOR (95%CI) = 4.32[1.58, 11.86], previous history of gastrointestinal disease AOR (95% CI) = 4.12[1.40, 12.65], history of abortion AOR (95% CI) = 6.23[2.24, 17.52] were factors significantly associated with hyperemesis gravidarum.

**Conclusion:**

In this study, the overall hospital proportion of hyperemesis gravidarum was high. History of gastrointestinal disease, previous history of urinary tract infection, history of hyperemesis gravidarum, and history of abortion were the major risk factors.

## Background

Hyperemesis gravidarum (HG) is severe nausea and repeated vomiting that prevents oral intake of food and leads to dehydration, ketoacidosis and nutritional deficiencies that may need hospitalization [1, 2]. The most commonly stated criteria for diagnosis of HG includes persistent vomiting not related to other causes (objective measure ketonuria on urine analysis), acid-base disturbances and electrolyte abnormalities, as well as weight loss of at least 5% from pre-pregnancy weight [3]. HG is usually limited to the first trimester and commonly resolve by the 20^th^ week of gestation, but around 10% of women have symptoms that continue throughout pregnancy [4].

Globally, nausea and vomiting of pregnancy NVP might occur around 90% of pregnancies, while HG affects 0.5–2% of all pregnancies [5]. The prevalence of HG varies with ethnicity. In the world, Asian and Middle Eastern ethnicities have higher rates of HG, even as high as 10.8% in a study reported by Fejzo et al. [6]. In Africa, studies conducted in Egypt and Northeast Nigeria revealed that the proportion of HG was 4.5% and 44.9% respectively [7, 8]. Studies conducted in Ethiopia Jimma university hospital, Addis Ababa three teaching hospitals and Arba Minch General Hospital stated that the proportion of HG was 4.8%, 4.4% and 8.2% respectively [9–11].

The complication of HG affects both mothers and foetuses. Most of the complications of HG were due to the effects of dehydration and starvation. Common maternal complications are neurologic complications, stress ulcers in the stomach, esophageal tear (Mallory-Weiss syndrome), jaundice, hepatic failure, convulsions and coma, and hypoprothrombinemia due to vitamin K deficiency, renal failure, and even leads to death [5, 12–14]. The fetal complications of HG are low birth weight, intrauterine growth restriction, preterm delivery, lower APGAR scores and fetal and neonatal death [15].

Common risk factors for HG are the previous history of HG, history of NVP in previous pregnancies women being less than 20 years, low-income level, low educational status, food aversions before pregnancy, multiple gestations, gastrointestinal disease, history of urinary tract infection, multiple pregnancy, and women having prior miscarriages, unplanned pregnancies, fetal abnormalities and primigravida women [15–20]. Furthermore, history of motion sickness, sensitivity to oral contraceptives, migraine headaches, allergies, peptic ulcer, family history of HG, high blood pressure, liver disease, kidney disease, over or under nutrition, previous molar pregnancy, pre-existing diabetes, and gastrointestinal disorders are increasing the risk of HG [7, 21–24].

Determine the proportion and associated factors of hyperemesis gravidarum among pregnant women have a specific importance for early detection and intervention to reduce the adverse perinatal outcome, hospitalization, physical, psychological, economic, and social burden of HG on women’s lives, through prevention and early initiation of intervention. However, very limited descriptive studies were conducted in Ethiopia especially the study area. Hence, this study was designed to determine the proportion of HG and associated factors among pregnant women admitted to Akesta general hospital, Amhara region, Ethiopia.

## Materials and methods

### Study design and setting

A study was conducted in Akesta general hospital, South Wollo zone, Amhara Region, Ethiopia. Akesta general hospital was constructed in 1990 and now gives health services to over one million people living in the catchment areas. Akesta town is located about 360km from Bahr Dar town which is the capital city of the Amhara region and 99 Km from Dessie town. It lies at 1917 meters above sea level. According to the 2012 E.C of Akesta general hospital and zonal health office reports the total hospital catchment’s population were 1,200,000 from that 598,800 were male and 601,200 were female. In Akesta town, there was one government hospital and 9 health centres. Within the previous two years around 1672 pregnant mothers were admitted to Akesta general hospital.

### Study population, sample size determination and procedures

The source population of this study was all pregnant women who were admitted to the obstetrics ward at Akesta general hospital. The study population of this study was all pregnant women who were admitted to the obstetrics ward in Akesta general hospital from September 1/2018 to August 30/2020. All pregnant women admitted to the obstetrics ward that completed their treatment, delivered or died after initiation of treatment in the hospital and who had complete data during the study period were included. The patient’s death on arrival was excluded.

The sample size was calculated by using a single population proportion formula, assuming 8,2% prevalence taken from a study conducted in Arba Minch general hospital [11], with a 95% confidence level, 3% marginal of error (d), and 10% non-response rate. The sample size was calculated with the following formula

n = [(Zα/2)2 ×P (1-P)]/d2 [25]

Where, p = 8.2% this is proportion of HG in Arba Minch general hospital.

Where Zα/2 at 95% confidence level = 1.96

d (margin of error) = 0.03

n = [(1.96)2 ×0.082(1–0.082)] / (0.03)2

= 322

When adding 10% non-respondents n = 355

This yields a sample size of 355.

First all admitted cases were selected from medical registration book during the study period and given unique medical registration numbers starting from least (1,2,3,4…N). A simple random sampling method was used to select the required number of case notes.

### Operational definition

#### Hyperemesis gravidarum

According to the latest American college of obstetricians and gynaecologists guidelines on nausea and vomiting during pregnancy (2015), still there is no single accepted definition of hyperemesis gravidarum. The most commonly cited criteria for diagnosis of hyperemesis gravidarum include persistent vomiting not related to other causes, an objective measure of acute starvation (ketonuria +1 and above on urine analysis), electrolyte abnormalities and acid-base disturbances, as well as weight loss. Weight loss is often cited as at least 5% loss of pre-pregnancy weight [3].

#### Nausea and vomiting of pregnancy

diagnosed when onset of nausea and vomiting is in the first trimester of pregnancy and other causes of nausea and vomiting have been excluded [26].

#### Grand multipara

was defined as parity of ≥5 with previous pregnancies of ≥28 weeks of gestation [27].

#### Abortion

Abortion is the expulsion of the conceptus before 28 completed weeks of gestation include both induced and spontaneous [28].

### Data collection and quality control

The structured checklist adopted from another study was used as a data collection instrument. Data about socio-demographic, medical, and obstetric factors were collected by a structured checklist. To control the quality of data, before data collection, training was given to data collectors and supervisors. Pre-test was conducted on 5% of the sample size at Dessie referral hospital. Possible modification of the checklist was made based on the result of pre-test. In addition close supervision was conducted by supervisors. Data were collected by 4 trained BSc midwives.

### Data management and analysis

The collected data were entered into Epi-Data version 7.3 and exported to the statistical package for social science (SPSS) version 23 for cleaning and analysis. Data cleaning was performed to check for accuracy, consistency, and missed values. Descriptive statistics were computed and reported in frequencies and percentiles.

Bivariable and multivariable logistic regression was used to assess the association between dependent and independent variables. All independent variables with a p-value less than 0.25 at bivariable analysis were entered into multivariable analysis to control for all possible confounders. P-value <0.05 was considered statistically significant and its strength was measured by using odds ratios 95% confidence intervals. Model fitness was assessed using the Hosmer-Lemeshow statistic test.

### Ethics approval and informed consent

Ethical clearance was obtained from the research ethical review committee of Wollo University, College of Medicine and Health Sciences, Institutional Research Ethics Review Board (CMHS841/04/13) and a permission letter was obtained from general Hospital. An informed consent was waived by the ethics committee of Wollo University, College of Medicine and Health Sciences, since the study was conducted through review of medical records. Further patients’ names were not recorded. All methods were performed in accordance with the relevant guidelines and regulations.

## Results

### Socio-demographic characteristics

A total of 355 pregnant women were involved in this study, with a response rate of 100%. Majority of the pregnant women 282 (79.4%) live in the rural area. More than three fourth 270 (76.1%) of the pregnant women were within the age group of 20–35 years, with a mean age of 26 years ± 5.8 SD. The majority of the respondents 348 (98%) of the pregnant women were married while 13(3.7%) had secondary education and above (Table 1).

**Table 1:** Socio-demographic characteristics of pregnant women, at Akesta General Hospital, Ethiopia, 2020.

Variables	Category	Hyperemesis gravidarum		Total n (%)	Chi-square p-value
		Yes n (%)	No n (%)		
Age	< 20 years	7(17.5)	56(17.8)	62(17.7)	0.21
	20–35 years	28(70.0)	242(76.8)	270(76.1)	
	>35 years	5(12.5)	17(5.4)	22(6.2)	
Religions	Muslim	38(95.0)	296(94.0)	334(94.1)	0.79
	Orthodox	2(5.0)	19(6.0)	21(5.9)	
Marital status	Married	39(97.5)	309(98.1)	348(98.0)	0.79
	Single	1(2.5)	6(1.9)	7(2.0)	
Residency	Urban	17(42.5)	56(17.8)	73(20.6)	<0.001
	Rural	23(57.5)	259(82.2)	282(79.4)	
Educational status	No formal education	11(27.5)	92(29.2)	103(29.0)	0.02
	Primary education	19(47.5)	174(55.2)	193(54.4)	
	Secondary education	5(12.5)	41(13.0)	46(13)	
	College and above	5(12.5)	8(2.5)	13(3.7)	
Occupation	Housewife	29(72.5)	279(88.6)	308(86.8)	0.017
	Employed	5(12.5)	10(3.2)	15(4.2)	
	Merchant	5(12.5)	21(6.7)	26(7.3)	
	Other	1(2.5)	5(1.6)	6(1.7)	

### Obstetric history

The proportion of HG in this study was 40 (11.3%) with 95% CI [8.2, 14.4]. Forty-four (12.4%) mothers had a history of nausea and vomiting in the previous pregnancy. The majority of 313(88.2%) current pregnancy was planned (Table 2).

**Table 2:** Obstetric history of pregnant women who were admitted at Akesta General Hospital, North East Ethiopia.

Variables	Category	Hyperemesis gravidarum		Total n (%)	Chi-square p-value
		Yes n (%)	No n (%)		
Previous history of HG	Yes	9(22.5)	4(1.3)	13(3.7)	<0.001
	No	31(77.5)	311(98.7)	342(96.3)	
Previous history of NVP	Yes	19(47.5)	25(7.9)	44(12.4)	<0.001
	No	21(52.5)	290(92.1)	311(87.6)	
Gravidity	Primigravida	8(20.0)	117(37.1)	125(35.2)	
	Multigravida	30(75.0)	186(59.1)	216(60.8)	<0.001
	Grand multigravida	2(5.0)	12(3.8)	14(4.0)	
Parity	Nullipara	14(35.0)	120(38.1)	134(37.7)	0.91
	Primipara	11(27.5)	93(29.5)	104(29.3)	
	Multipara	15(37.5)	102(32.4)	117(34.0)	
History of abortion	Yes	12(30.0)	17(5.4)	29(8.2)	<0.001
	No	28(70.0)	298(94.6)	326(91.8)	
History of molar pregnancy	Yes	1(2.5)	4(1.3)	5(1.4)	<0.001
	No	32(80.0)	301(95.6)	333(98.3)	
	Don’t Know	7(17.5)	10(3.2)	17(4.8)	
Have prior infant weight <2500 gram	Yes	0(0.0)	9(2.9)	9(2.5)	0.43
	No	37(92.7)	291(92.4)	328(92.4)	
	Don’t Know	3(7.5)	15(4.8)	18(5.1)	
Previous congenital anomaly foetus	Yes	3(7.5)	10(3.1)	13(3.7)	0.004
	No	37(92.5)	305(96.9)	342(96.3)	
The status of the current pregnancy	Planned	33(82.5)	280(88.9)	313(88.2)	0.24
	Unplanned	7(7.5)	35(11.1)	42(11.8)	
Gestational age	<12 Weeks	34(85.0)	6(1.9)	40(11.3)	<0.001
	≥12 Weeks	6(15.0)	309(98.1)	315(88.7)	
Current pregnancy interval	<24 months	13(41.9)	27(13.6)	40(17.4)	<0.001
	≥24 months	18(58.1)	172(86.4)	190(82.6)	
Current pregnancy type	Singleton	34(85.0)	286(90.8)	320(90.1)	0.25
	Multiple	6(15.0)	29(9.2)	35(9.9)	

### Medical and family history

Forty (11.3%), and 28(7.9%), of women had a history of urinary tract infections, and gastrointestinal disease respectively. Twenty-three (6.5%) had a history of pregnancy-induced hypertension (Table 3).

**Table 3:** Medical and family history of women with HG, at Akesta General Hospital, North East Ethiopia.

Variables	Category	Hyperemesis gravidarum		Total n (%)	
		Yes n (%)	No n (%)		
Diagnosed with the hyperthyroid disorder	Yes	1(2.5)	0(0)	1 (.3)	0.15
	No	36(90.0)	299(94.6)	335 (94.4)	
	Don’t know	3(7.5)	16(5.1)	19 (5.4)	
Have a history of pregnancy-induced hypertension	Yes	1(2.5)	22(7.0)	23 (6.5)	0.389
	No	38(35.0)	290(92.0)	328 (92.4)	
	Don’t know	1(2.5)	3(1.0)	4 (1.1)	
Have pregnancy-induced diabetes before the current pregnancy	Yes	0(0.0)	4(1.3)	4 (1.1)	0.038
	No	38(95.0)	309(98.1)	347 (97.7)	
	Don’t know	2(5.0)	2(0.6)	4 (1.1)	
Have a migraine headache?	Yes	8(20.0)	38(12.1)	46 (13.0)	0.155
	No	32(80.0)	277(87.9)	309 (87)	
Have a history of GI diseases	Yes	13(32.5)	16(5.1)	29 (7.9)	<0.001
	No	27(67.5)	299(94.9)	327 (92.1)	
Have a history of UTIs?	Yes	14(35.0)	26(8.3)	40 (11.3)	<0.001
	No	26(65.0)	289(91.7)	315 (88.7)	

### Factors associated with hyperemesis gravidarum

The odds of hyperemesis gravidarum were 10.9 times higher among women who had previous history of HG compared with women who had no previous history of HG AOR (95%CI) = 10.9[2.46, 48.44]. The odds of hyperemesis gravidarum were 4.32 times higher among women who had previous history of UTI compared with women who had no previous history of UTI AOR (95%CI) = 4.32[1.58, 11.86]. The odds of hyperemesis gravidarum were 4.12 times higher among women who had previous history of gastrointestinal disease compared with women who had no history of gastrointestinal disease AOR (95% CI) = 4.12[1.40, 12.65] (Table 4).

**Table 4:** Factors associated with hyperemesis gravidarum among pregnant women at Akesta General Hospital, North East Ethiopia.

Variables	Categories	Hyperemesis gravidarum		COR (95%CI)	AOR (95%CI)
		Yes	No		
Previous history of HG	No	31	311	1	1
	Yes	9	4	22.6[6.6,77]	10.9[2.46,48.44]
History of abortion	No	28	298	1	1
	Yes	12	17	7.5[3.3,17.3]	6.23[2.24,17.52]
History of GI diseases	No	26	297	1	1
	Yes	14	18	8.85[3.6,19.4]	4.12[1.40, 12.65]
History of UTI	No	26	289	1	1
	Yes	14	26	5.99[2.8,12.9]	4.32[1.58,11.86]

## Discussion

An increased prevalence of HG is a public health concern in the world. Because, it is severe and persistent that may need hospitalization. This study aimed to determine the proportion and associated factors of HG among women who were admitted in Akesta general hospital. The finding of this study revealed that previous history of HG, history of abortion, history of gastrointestinal disease, history of UTI and gravidity were significantly associated with HG.

The proportion of HG in this study was 11.3%, with 95%CI [8.2–14.4]. This finding was consistent with the study conducted in Arba Minch (8.2%) and China (10.8%) [6, 11]. However, the proportion of this study was higher than studies conducted in Egypt (4.5%), Addis Ababa (4.4%), and Jimma town (4.8%) [7, 9, 10]. This inconsistency might be due to the difference in study design, sample size and the definition used to diagnose HG. Still there is no single accepted definition of hyperemesis gravidarum. Furthermore, most of the catchment areas of this hospital are rural. Pregnant women who live in rural areas are of poor socioeconomic status and are exposed to stress that increases the severity of nausea and vomiting. While this finding was lower than the study conducted in North-East Nigeria (44.9%) [8]. This difference might be due to differences in study design and study population.

Women who had previous history of HG were eleven times at higher odds of developing HG as compared to their counterparts. This finding was consistent with another study that was conducted in Germany [29]. This may be due to the presence of a recurrent risk factor in the patients. Besides the stress increase during current pregnancy due to fear of previous HG hence, stress is one of the aggravating factors for HG.

This study revealed that pregnant mothers with a previous history of gastrointestinal disease were four times more likely to develop HG when compared with those without previous history of gastrointestinal disease. This finding was consistent with other studies conducted in Egypt and Jimma [7, 9]. This might be due to the presence of a previous history of GI disease in the patients which increases the severity of nausea and vomiting. When the women have previous history of gastrointestinal disease and not completely treat this disease increase the severity of nausea and vomiting in the current pregnancy.

The odds of developing HG were six times higher among women who had history of abortion as compared to no had history of abortion. This finding is also consistent with another study conducted in Israel [30]. The possible explanation could be related to psychological factors (fear of pregnancy due to past failures). This indicates that a previous history of abortion was one of the risk factors to develop HG.

Finally, the odds of developing hyperemesis gravidarum were four times higher among pregnant women who had a previous history of UTI as compared to those who had not a history of UTI. This finding is parallel with other studies conducted in Jimma town [9]. This might be due to when there is an infection aggravating the severity of nausea and vomiting. During the treatment of UTI not completely treated still on-going at the time of current pregnancy increase the severity of nausea/vomiting.

This result interpreted with the following limitations, its retrospective cross sectional design. The most important limitation in chart review data is the possibility that some severe vomiting cases may not have been diagnosed because of incomplete data. Thus, some HG cases may have been missed.

## Conclusion

The proportion of hyperemesis gravidarum at Akesta general hospital was high. The independent associated factors of HG were previous history of HG, history of gastrointestinal disease, history of abortion and history of UTI. Healthcare providers should give health education and counseling service concerning about pre pregnancy care. Before pregnancy, women should be screened and treated for UTI and gastrointestinal disease.

## Supporting information

S1 Dataset (SAV)

## Acronym/abbreviation

AOR: Adjusted odds Ratio
CI: Confidence interval
COR: Crude odds Ratio
HG: Hyperemesis Gravidarum
NVP: Nausea and Vomiting of Pregnancy
UTI: Urinary tract infection
